# HEALTHCARE SERVICES UTILIZATION IN ELDERLY RESIDENTS OF A PERUVIAN HIGHLAND COMMUNITIES: AUNQUI-ANDES STUDY

**DOI:** 10.1093/geroni/igad104.3384

**Published:** 2023-12-21

**Authors:** Nelson Luis Cahuapaza-Gutierrez, Kamyla M Olazo-Cárdenas, Diego Urrunaga-Pastor, Fernando M Runzer-Colmenares

**Affiliations:** Universidad Científica del Sur, Lima, Lima, Peru; Universidad Científica del Sur, Lima, Lima, Peru; Universidad Científica del Sur, Lima, Lima, Peru; Universidad Científica del Sur, Lima, Lima, Peru

## Abstract

It is estimated that approximately one out of every three Peruvians does not utilize healthcare services when facing an ailment. Nevertheless, this frequency could be higher in highland regions, given the limited availability of healthcare services. The aim was to estimate the prevalence of non-utilization of healthcare services among elderly residents of a Peruvian highland community during 2022. We conducted an analytical cross-sectional study in the district of Totos (average altitude of 3286 meters above sea level) in the Ayacucho region of Peru during 2022. A census sampling method was employed, including residents aged 60 and older in Totos. Non-utilization of healthcare services was defined as a participant not seeking healthcare services when facing a health issue. The current study was approved by the Institutional Research Ethics Committee of Universidad Científica del Sur. We evaluated 272 elderly adults with an average age of 74 years, of which 60.3% were female, 62.5% were cohabiting or married, 82.3% had not completed primary education, and 65.4% reported being beneficiaries of a social program for economic support. We found that 28.3% did not use healthcare services, while 44.9% reported using traditional medicine, and 13.2% practiced self-medication. Participants reported that the nearest health center to their residence was, on average, 55 minutes away. We found that approximately one out of every five elderly adults did not use healthcare services. It is necessary to reduce cultural barriers to increase healthcare service utilization, as well as to enhance their availability for elderly individuals living at high altitudes.

